# MicroRNA networks regulated by *all-trans* retinoic acid and Lapatinib control the growth, survival and motility of breast cancer cells

**DOI:** 10.18632/oncotarget.3759

**Published:** 2015-04-18

**Authors:** James Neil Fisher, Mineko Terao, Maddalena Fratelli, Mami Kurosaki, Gabriela Paroni, Adriana Zanetti, Maurizio Gianni, Marco Bolis, Monica Lupi, Anna Tsykin, Gregory J. Goodall, Enrico Garattini

**Affiliations:** ^1^ Laboratory of Molecular Biology, IRCCS-Istituto di Ricerche Farmacologiche “Mario Negri”, Milano, Italy; ^2^ Department of Oncology, IRCCS-Istituto di Ricerche Farmacologiche “Mario Negri”, Milano, Italy; ^3^ Centre for Cancer Biology, SA Pathology, Adelaide, Australia and Department of Medicine, University of Adelaide, Adelaide, Australia

**Keywords:** retinoic acid, microRNA, breast cancer, lapatinib, network analysis

## Abstract

*SKBR3*-cells, characterized by *ERBB2*/*RARA* co-amplification, represent a subgroup of *HER2*^+^ breast-cancers sensitive to *all-trans* retinoic acid (ATRA) and Lapatinib. In this model, the two agents alone or in combination modulate the expression of 174 microRNAs (miRs). These miRs and predicted target-transcripts are organized in four interconnected modules (*Module-1* to *-4*). *Module-1* and *Module-3* consist of ATRA/Lapatinib up-regulated and potentially anti-oncogenic miRs, while *Module-2* contains ATRA/Lapatinib down-regulated and potentially pro-oncogenic miRs. Consistent with this, the expression levels of *Module-1/-3* and *Module-2* miRs are higher and lower, respectively, in normal mammary tissues relative to ductal-carcinoma-*in-situ*, invasive-ductal-carcinoma and metastases. This indicates associations between tumor-progression and the expression profiles of *Module-1* to *-3* miRs. Similar associations are observed with tumor proliferation-scores, staging, size and overall-survival using TCGA (The Cancer Genome Atlas) data. Forced expression of *Module-1* miRs, (miR-29a-3p; miR-874-3p) inhibit *SKBR3*-cell growth and *Module-3* miRs (miR-575; miR-1225-5p) reduce growth and motility. *Module-2* miRs (miR-125a; miR-193; miR-210) increase *SKBR3* cell growth, survival and motility. Some of these effects are of general significance, being replicated in other breast cancer cell lines representing the heterogeneity of this disease. Finally, our study demonstrates that HIPK2-kinase and the PLCXD1-phospholipase-C are novel targets of miR-193a-5p/miR-210-3p and miR-575/miR-1225-5p, respectively.

## INTRODUCTION

The estrogen receptor-negative and HER2^+^ *SKBR3* cell line is representative of a recently identified breast cancer subtype characterized by co-amplification of the genes coding for the HER2 membrane receptor (*ERBB2*) and the RARα nuclear retinoid receptor (*RARA*), respectively [1]. This breast cancer subtype is predicted to be extremely sensitive to the anti-tumor action of *all-trans* retinoic acid (ATRA), the active metabolite of vitamin A [1, 2]. In *SKBR3* cells, simultaneous targeting of RARα with ATRA and HER2 with Lapatinib results in synergistic anti-tumor responses [1]. The molecular determinants at the basis of this anti-tumor activity need to be identified.

MicroRNAs (miRs) are short regulatory RNAs controlling the stability and translation of target transcripts [3]. MiRs control numerous processes in the neoplastic cell [4, 5] and they can be characterized as oncogenic or anti-oncogenic [4–10]. The *SKBR3* model provides a unique opportunity to establish whether miRs play any role in the cell-autonomous anti-tumor responses triggered by ATRA and Lapatinib. A potential role of these regulatory RNAs in the anti-tumor action of ATRA is suggested by studies performed in various cellular contexts [11–25], although very little information is available in the setting of breast cancer cells [26, 27]. In the estrogen receptor-positive *MCF-7* cell line, ATRA causes up-regulation of a single miR, i.e. miR-21 [27]. Similarly, there is limited experimental evidence on the links between miRs and Lapatinib anti-tumor activity [28–33].

Here, we demonstrate that ATRA and Lapatinib, alone or in combination, modify the miR expression profile of *SKBR3* cells substantially. Some of the miRs up- or down-regulated by the two agents control the growth, survival and motility of *SKBR3* cells and other cell lines representative of breast cancer heterogeneity. The regulated miRs and predicted target transcripts are organized in four highly interconnected functional modules. The miR expression fingerprints defined by the four modules are of general interest, being associated with breast cancer progression and prognosis.

## RESULTS

### Multiple anti-tumor responses in the SKBR3 cell line by pharmacological targeting of HER2 and RARα

**Figure 1:**
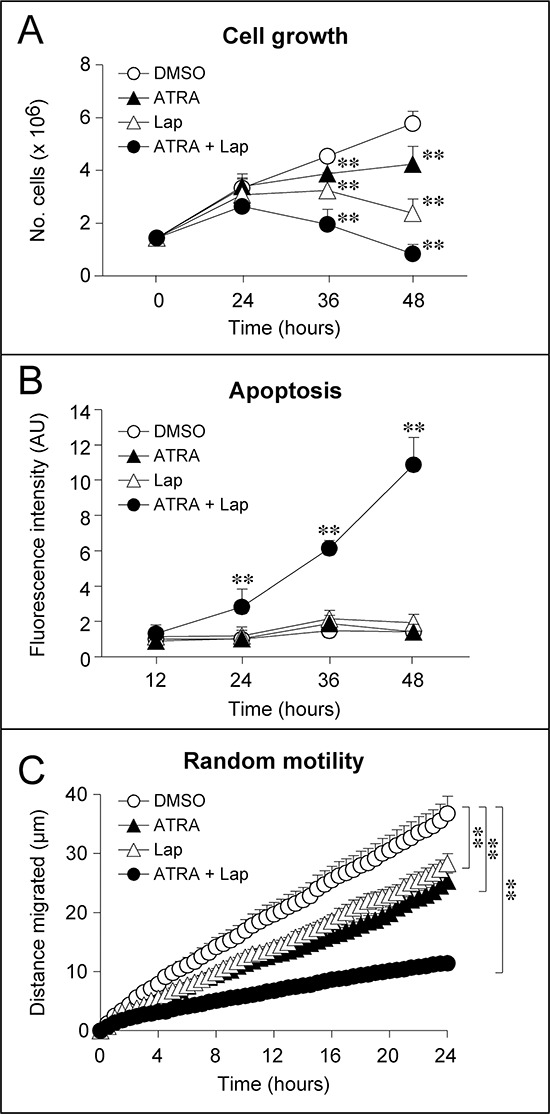
Effect of ATRA and Lapatinib alone or in combination on the growth, apoptotic response and motility of SKBR3 cells *SKBR3* cells were treated with vehicle (DMSO), Lapatinib (100 nM), ATRA (100 nM) or Lapatinib+ATRA for the indicated amount of time. **A.** Time course for the growth inhibitory effects of ATRA and/or Lapatinib. Viable cells were counted following incubation with Trypan Blue. The results are the mean ± SD of 3 culture dishes. **Significantly different relative to vehicle treated cells (*p* < 0.01, Student's *t*-test). **B.** Apoptotic response induced by ATRA and/or Lapatinib. The apoptotic response was evaluated by measuring the levels of caspase-3/7 activity on cell extracts obtained at the indicated time points using a fluorescent substrate. The results are the mean ± SD of 3 culture dishes. **Significantly different relative to vehicle treated cells (*p* < 0.01, Student's *t*-test). **C.** Effects of ATRA and/or Lapatinib on random cell motility. Random cell motility was evaluated by time-lapse microscopy. Each curve represents the motility of at least 40 cells. Each value is expressed as the mean ± SE of the distance migrated by each individual cell. **Significantly different (*p* < 0.01, two-way ANOVA Bonferroni post-test).

Targeting of HER2 with Lapatinib and RARα with ATRA results in a number of anti-tumor responses. Both ATRA and Lapatinib cause inhibition of *SKBR3* cell growth, which is remarkably enhanced upon simultaneous exposure to the two compounds (Fig. 1A). In addition, a strong apoptotic response is evident upon co-treatment with ATRA and Lapatinib (ATRA+Lapatinib), as indicated by measurement of caspase-3/7 activity (Fig. 1B). This is observed in conditions where treatment with ATRA or Lapatinib alone does not result in apoptosis. Growth inhibition and programmed cell death are accompanied by signs of epithelial and lactogenic differentiation which are visible upon treatment with ATRA and to a greater extent by ATRA+Lapatinib [1]. Finally, challenge with the retinoid or the HER2 tyrosine kinase inhibitor decreases random-motility, a process associated with the invasive and metastatic behavior of cancer cells (Fig. 1C). Also in this case, co-treatment with ATRA and Lapatinib enhances the activity of the single components of the mixture. Altogether, our results indicate that ATRA and Lapatinib alone or in combination exert direct effects of therapeutic relevance on the neoplastic cell.

### Perturbations of miR expression by ATRA and/or Lapatinib

**Figure 2:**
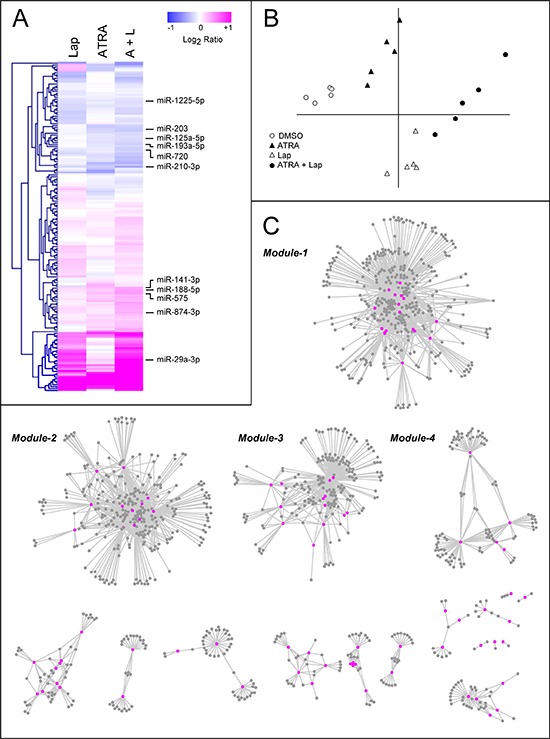
Modulation of miR expression following treatment of SKBR3 cells with ATRA and/or Lapatinib and network analysis of the miR/target-mRNA interactions *SKBR3* cells were treated with vehicle (DMSO), Lapatinib (100 nM), ATRA (100 nM) or ATRA+Lapatinib for 36 hours. RNA was extracted and used for the determination of the miR expression profiles, using a miR microarray platform. **A.** Heat map of the miR expression profiles. The data are expressed as the Log_2_ of the miR expression ratio calculated for Lapatinib vs. vehicle (Lap), ATRA vs. vehicle (ATRA) and ATRA+Lapatinib vs. vehicle (A+L). Each result is the mean of 5 biological replicates. The miRs used for the validation and over-expression studies are indicated on the right. **B.** The Principal Component Analysis (PCA) of the miR expression levels observed in *SKBR3* cells treated with ATRA, Lapatinib or the combination of the two compounds is shown. **C.** The panel illustrates the network representing the miR/target-mRNA interactions predicted by the MAGIA algorithm. The network consists of several modules and the four most inter-connected modules are indicated (*Module-1* to *-4*). Cytoscape was used to build the network. The magenta nodes represent miRs, while the grey nodes represent miR target-mRNAs.

To gain insights into the significance of miRs [20–25] for the responses triggered by the two anti-tumor compounds, we determined the expression profiles of these small regulatory RNAs in *SKBR3* cells following challenge with vehicle, ATRA, Lapatinib and ATRA+Lapatinib for 36 hours. Of the 1, 205 miRs represented on the microarray [27], 330 show detectable expression levels in at least one of the experimental conditions considered. One hundred and seventy four miRs are up- or down-regulated by ATRA, Lapatinib or ATRA+Lapatinib in a consistent and significant manner (Fig. 2A and Suppl. Table S1). Principal component analysis (PCA) of the data indicates that Lapatinib exerts a larger overall effect on the expression of these regulatory RNAs than ATRA (Fig. 2B). The combination of ATRA and Lapatinib results in a further quantitative and qualitative modulation of the miR expression profiles. The microarray data are validated and confirmed for 8 miRs by real-time quantitative PCR (qPCR) (Suppl. Fig. S1A). We took two representative miRs modulated predominantly by Lapatinib (miR-29a-3p) and by both ATRA and Lapatinib (miR-210-3p) for further time-course studies. The up-regulated miR-29a-3p, and the down-regulated miR-210-3p were selected based on the following criteria: a) high basal expression level; b) significant fold change caused by ATRA+Lapatinib; c) high correlation *r*^2^ value between the qPCR and the microarray data. Lapatinib causes an early induction of miR-29a-3p with a maximal effect at 20 hours, while miR-210-3p down-regulation by ATRA and/or Lapatinib is gradual (Suppl. Fig. S1B).

### Coordinated regulation of common target-transcripts by multiple miRNAs

ATRA, Lapatinib and ATRA+Lapatinib cause significant perturbations of the miR and gene-expression profiles [1] (E-MEXP-3192; http://www.ebi.ac.uk/arrayexpress) in *SKBR3* cells (Suppl. Fig. S2). To identify potential miR targets from the 3, 144 mRNAs altered by the treatments applied and to study the organization of the predicted miR/target-transcript interactions, we used two independent criteria (MAGIA webtool, http://gencomp.bio.unipd.it/magia): 1) Direct target transcripts of the 174 miRs regulated by ATRA and/or Lapatinib must be predicted by at least one of the three algorithms considered; 2) Following treatment with ATRA, Lapatinib and ATRA+Lapatinib, the expression profiles of the candidate target mRNAs and the putative modulating miR(s) have to be inversely correlated (Spearman correlation index < −0.9). This results in 2, 829 possible miR/target-mRNA interactions involving 89 distinct miRs and 1, 116 mRNAs (Suppl. Table S2).

**Figure 3:**
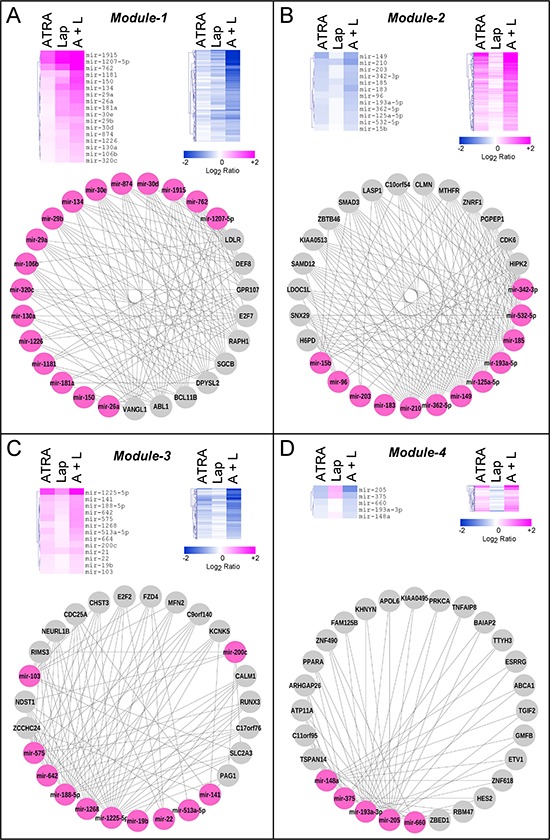
Expression pattern and connectivity of the miRs and corresponding target-mRNAs in the four modules The panels show the heat-maps of miR (upper-left) and miR target-mRNAs (upper-right) expression in *SKBR3* cells challenged with ATRA, Lapatinib or ATRA+Lapatinib for 36 and 48 hours, respectively. The data are expressed as the Log_2_ of the miR expression ratio calculated for Lapatinib vs. vehicle (Lap), ATRA vs. vehicle (ATRA) and ATRA+Lapatinib vs. vehicle (A+L). A network representation (degree sorted circle layout) of the most connected miRs (magenta) and miR target-mRNAs (grey) is illustrated at the bottom of each panel. Panels **A–D.** illustrate the miRs and target-mRNAs belonging to *Module-1* to *-4*, respectively. Each target-mRNA contained in the Network diagram of *Module-1* and *-3* are predicted to be regulated by at least 10 of the 17 miRs as well as at least 5 of the 13 miRs present in the two modules, respectively. Each target-mRNA contained in the Network diagram of *Module-2* and *-4* are predicted to be regulated by at least 8 of the 12 miRs as well as at least 2 of the 5 miRs present in the two modules, respectively.

Network analysis of these interactions reveals four highly interconnected miR and target-mRNA sub-networks, which we define as *Module-1* to *Module-4* (Fig. 2C and Suppl. Table S3). The four modules consist of miRs which are predicted to control the expression of multiple and common target-mRNAs (Fig. 3A–3D, lower schemes), a feature which is defined by the calculated degree of connectivity (Table 1). For each node (miR or target-mRNA) in the network, the degree of connectivity is the number of relations (edges) to the other nodes. For miRs, this is the number of predicted target-mRNAs, while, for mRNAs, it is the number of miRs which are predicted to regulate their expression. *Module-1* is characterized by the largest number of miRs and the highest degree of connectivity, followed by *Module-2, -3* and *-4* in sequence. Both *Module-1* and *-3* contain miRs and putative target-mRNAs which are up- and down-regulated, respectively, by the treatments applied. *Module-2* is representative of miRs and target-mRNAs which are down-regulated and up-regulated, respectively, in our experimental conditions. The expression pattern of *Module-4* miRs is more complex, as ATRA and Lapatinib alone have opposing effects on these regulatory RNAs. In *Module-1*, miR up-regulation and target-mRNA down-regulation are predominantly afforded by Lapatinib and they are enhanced by ATRA+Lapatinib (Fig. 3A). In *Module-2*, ATRA is the predominant regulator of miRs and target-mRNAs (Fig. 3B). *Module-3* consists of miRs up-regulated and mRNAs down-regulated predominantly by ATRA alone or in combination with Lapatinib (Fig. 3C). *Module-4* contains miRs down-regulated by ATRA and up-regulated by Lapatinib as well as target-transcripts with an opposite expression pattern (Fig. 3D).

**Table 1:** MiR/mRNA composition and calculated degree of connectivity for the identified network

	No. miRs	No. mRNAs	No. interactions	Average *DOC*	Average miR *DOC*	Average mRNA *DOC*
***Whole Network***	89	1, 118	2, 833	4.69	31.83	2.53
***Module-1***	17	332	1, 101	6.31	64.76	3.32
***Module-2***	12	276	805	5.59	67.08	2.92
***Module-3***	13	176	426	4.51	32.77	2.42
***Module-4***	5	94	124	2.51	24.80	1.32
***No Module***	42	240	377	2.67	8.98	1.57

The miRs and mRNAs modulated by ATRA and/or Lapatinib in *SKBR3* cells were subjected to Network analysis which led to the identification of the four highly inter-connected modules (*Module-1*/*Module-4*). *DOC* = Degree of connectivity; No Module = miRs and mRNAs which fall outside *Module-1* to *Module-4*.

Overall our data suggest that ATRA, Lapatinib and ATRA+Lapatinib regulate the expression of four groups of interconnected miRs, which, in turn, modulate gene-product networks in a concerted manner.

### Relative importance of *Module-1* to *-4* in the cellular processes associated with tumor progression

Enrichment analysis performed on the whole panel of miR target-genes contained in *Module-1 to -4* (Suppl. Table S3) results in the identification of eleven process networks, which directly or indirectly control cell-cycle, apoptosis and cell motility/invasiveness in at least one of the modules (false discovery rate, FDR < 0.05, Table 2). *Module-1* contains gene networks controlling mitosis and cell-cycle (G1- to S-phase progression) as well as blood vessel morphogenesis. *Module-2* is involved in the growth factor regulation of G1- to S-phase transit and epithelial to mesenchymal transition (EMT), which is fundamental for cancer stem cell homeostasis and tumor cell motility/invasiveness [34–37]. *Module-3* is implicated in the WNT signaling pathway, which stimulates EMT [38–41]. In addition, the module is relevant for the process of apoptosis and contains the same networks of cell-cycle genes belonging to *Module-1*. Interestingly, the action of *Module-1* and *Module-3* miRs, which show similar regulation patterns, impinges on analogous processes. *Module-4* is enriched in pathways related to cell motility/invasiveness, such as cytoskeleton-rearrangement and cell-adhesion.

**Table 2:** Enrichment analysis of the functional processes regulated by *Module-1* to *-4* miR target genes

Process Networks	Total	*Module-1*		*Module-2*		*Module-3*		*Module-4*	
	*NOB*	*NOB*	*FDR*	*NOB*	*FDR*	*NOB*	*FDR*	*NOB*	*FDR*
Cell cycle_Mitosis	179	30	**1.38E-15**	2	0.962	9	**0.020**	3	0.418
Cell cycle_G1-S	163	19	**2.43E-7**	6	0.283	8	**0.028**	2	0.601
Signal transduction_WNT signaling	177	8	0.355	6	0.293	12	**0.001**	2	0.631
Cytoskeleton_Regulation of cytoskeleton rearrangement	183	6	0.827	4	0.744	6	0.245	8	**0.005**
Development Neurogenesis_Synaptogenesis	180	2	0.997	4	0.732	10	**0.013**	2	0.631
Cell cycle_G1-S Growth factor regulation	195	3	0.997	11	**0.029**	7	0.145	3	0.458
Development_Neurogenesis_Axonal guidance	230	3	0.997	2	0.966	3	0.935	8	**0.012**
Development_Blood vessel morphogenesis	228	15	**0.006**	7	0.310	1	0.965	3	0.501
Development_EMT_Regulation of epithelial-to-mesenchymal transition	225	11	0.186	11	**0.029**	6	0.371	3	0.501
Cell adhesion_Integrin-mediated cell-matrix adhesion	214	3	0.997	2	0.962	6	0.328	7	**0.029**
Apoptosis_Apoptotic nucleus	159	8	0.272	6	0.283	8	**0.027**	1	0.783

All the miR target-mRNAs (Total) and the miR target-mRNAs present in *Module-1* through *Module-4* were analyzed for significant enrichment in the regulation of functional processes (Process Networks). The table shows enrichment analysis of the target genes in each Module on Metacore-annotated process networks (http://thomsonreuters.com/metacore/). Each process represents a pre-set network of protein interactions defined and annotated in the Metacore platform. For each module and for all the process networks significantly enriched in 1 of the modules, the table reports the FDR (false discovery rate), the number and the list of object networks. The grey boxes indicate eleven process networks showing significant enrichment (FDR < 0.05) in at least 1 module. NOB = Network objects.

It remains to be established whether the enrichment of miR target-mRNAs involved in the control of key aspects of the cancer cell homeostasis, such as growth, survival, EMT and invasiveness mediates or is simply the consequence of the anti-tumor responses triggered by ATRA and/or Lapatinib in *SKBR3* cells.

### The miR fingerprints associated with the anti-tumor action of ATRA+Lapatinib are related to breast cancer progression

Network and enrichment analyses indicate that miRs belonging to *Module-1* through *-4* act in a coordinated manner and control the expression of distinct target-mRNA sets characterized by general relevance for the growth, survival and motile/invasive behavior of breast cancer cells. To evaluate whether the relevance of the miR fingerprints defined by the four modules goes beyond the specific cellular model used in our studies, we investigated the expression patterns of the miRs constituting *Module-1* to *-4* in the clinical context of breast cancer.

**Figure 4:**
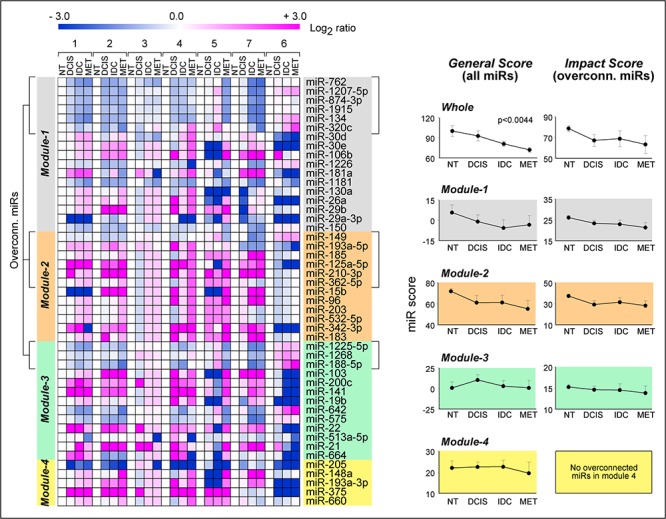
Association of *Module1-4* miR profiles with breast cancer progression Using the GSE38867 from the NCBI Gene Expression Omnibus (http://www.ncbi.nlm.nih.gov/gds), the expression profiles of *Module-1* to *-4* miRs were determined at various stages of disease progression (NT = normal tissue; DCIS = ductal carcinoma *in situ*, IDC = invasive ductal carcinoma; MET = metastasis) in 7 breast cancer patients. Left: Heat-map of the miR expression values (Log_2_ ratios relative to the corresponding NT samples). The over-connected miRs (miRs with degree >50, i.e. those with more than 50 predicted target-mRNAs) are indicated in square brackets. Right: The panels report two types of similarity scores: a *General Score* based on all the miRs and an *Impact Score* based on the subset of over-connected miRs. Both score types were calculated using all 47 miRs (*Whole*) or the miRs contained in each module. Each point represents the mean ± SE of the score calculated in the 7 patients at the four stages. The indicated *p*-value is calculated by one-way ANOVA.

In a first set of analyses, we evaluated the expression profiles of *Module-1* to *-4* miRs in a small and unique dataset (GSE38867) for which miR expression data are available during different phases of disease progression (ductal carcinoma *in-situ*, DCIS; invasive ductal carcinoma, IDC; metastasis, MET) and in matched samples representing normal mammary tissue (NT). The heat-maps in DCIS, IDC and MET *vs*. NT (Fig. 4, left-panel) are highly suggestive of an inverse relationship between the expression profiles of miRs up- or down-regulated by ATRA and/or Lapatinib in *SKBR3* cells and the corresponding profiles observed in DCIS, IDC and MET. In other words, the levels of *Module-1* and *-3* miRs up-regulated in response to the anti-cancer agents are generally lower in the three phases of tumor progression than in NT in six out of seven cases. Similarly, the expression of *Module-2* and *-4* miRs, which are predominantly down-regulated by the two drugs, tend to be higher in the three phases of tumor progression than in NT. This pattern is most evident when only the over-connected miRs (degree >50) are considered. To confirm this relationship in a quantitative manner, we defined similarity-scores based on all the miRs (*General Score*) or the subset of over-connected miRs (*Impact Score*). The *General Score* and the *Impact Score* were calculated for the whole signature (*Whole*) and for each module separately (Fig. 4, right-graphs). All the calculated scores show a general trend towards a decrease along disease progression. One-way ANOVA demonstrates significance in the case of the *General Score* determined for the whole signature. Interestingly, the greatest contribution to the observed decrease from NT to MET is provided by miRs belonging to *Module-1* and *Module-2*.

**Figure 5:**
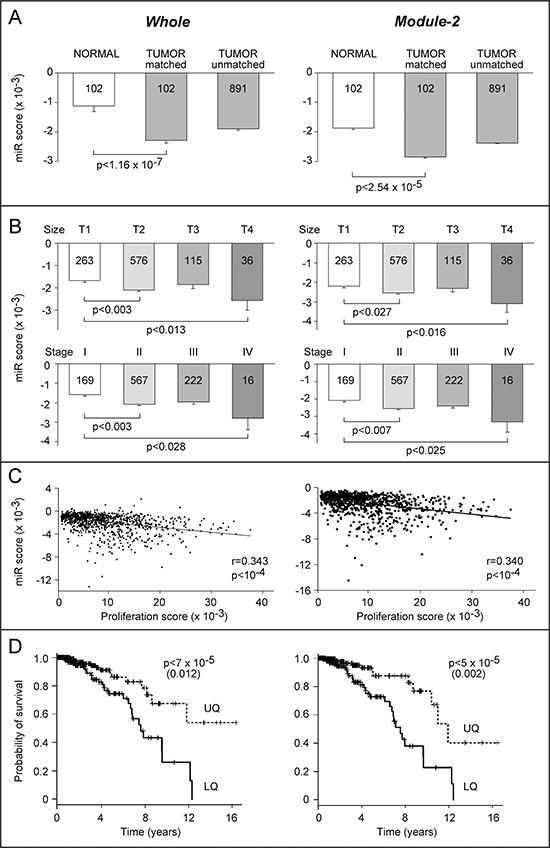
Association of *Module-1* to *-4* miR profiles with clinical indicators in the breast cancer TCGA dataset **A.** The *Impact Score* for the *Whole* (left) and *Module-2* (right) miR signatures was calculated for 102 normal mammary tissue samples and matched breast cancer samples as well as 891 unmatched tumors present in the TCGA dataset. The values are the mean ± SE of the indicated number of patients. The *p*-values refer to paired *t*-tests. **B.** The panel reports the same scores as in A after stratification of the tumor samples for size (T1-T4, upper graphs) and stage (I-IV, lower graphs). The values are the mean ± SE of the indicated number of patients. The *p*-values refer to Tukey's tests following one-way ANOVA. **C.** The *Impact Score* for the *Whole* (left) and *Module-2* (right) miR signatures were plotted against the PAM50 proliferation score. The *r* Pearson correlation values and the statistical significance are indicated. **D.** Kaplan-Meier overall survival curves for patients falling within the upper (dotted line, UQ) and lower (solid line, LQ) quartiles of the *Whole* and *Module-2* scores. The indicated *p*-values refer to the log-rank test. The values after correction for tumor size and stage with Cox proportional hazard test are shown in parenthesis.

The results obtained were validated and extended using the Cancer Genome Atlas (TCGA) dataset, which includes a cohort of 993 breast cancer cases. For 102 of these patients the miR expression profiles were determined in tumors and matched normal mammary tissues. A significantly higher *Impact Score* value is observed in normal relative to tumor tissues in matched patients (Fig. 5A). This association is further supported in the remaining 891 tumors present in the database. Indeed, this set and the original group of 102 tumors are characterized by similar scores. The *Impact Score* value is also higher in normal relative to tumor-matched and tumor-unmatched samples, if only the miR fingerprint corresponding to *Module-2* is considered. This is consistent with the idea that an effective anti-tumor treatment, such as the combination of ATRA and Lapatinib, modifies the miR profile of sensitive breast cancer cells to make it more similar to that observed in normal mammary cells. To support the relationship between our miR fingerprints and disease progression, we performed the same type of analysis after stratification of the tumors according to size, stage and cell-proliferation score. Tumors characterized by a small size (T1) show an *Impact Score* which is higher than the one observed in tumors characterized by progressively larger sizes (T2, T3 and T4) (Fig. 5B, upper-graphs). Once again a similar effect is evident if the analysis is restricted to the *Module-2 Impact Score*. Similarly, stage I show a higher *Impact Score* than stage II-IV tumors (Fig. 5B, lower-graphs). Finally, inverse correlations between *Whole* and *Module-2 Impact Scores* and the proliferation scores of each tumor sample are evident (Fig. 5C).

To evaluate whether the miR fingerprints identified by *Module-1* to *-3* have prognostic significance, we stratified the cases present in the TCGA dataset in quartiles according to their miR similarity scores. The cases in the upper quartile show an overall survival which is significantly more extended than the one observed in the lower quartile (Fig. 5D). The same type of analysis demonstrates that *Module-2* is the only module characterized by a miR fingerprint associated with a better prognosis in terms of overall survival. The difference in survival maintains significance after multivariate COX proportional hazard analysis with size and stage.

### Functional significance of selected *Module-1* and *Module-3* miRs in SKBR3 cells

The *Impact Scores* calculated for *Module-1* and *Module-3* miRs reveal no significant associations with the tumor relative to the normal mammary gland tissue in the TCGA dataset. Although the integrated expression profiles of the most-interconnected miRs belonging to *Module-1* and *Module-3* are not associated with tumor progression, the finding does not rule out the possibility that single members of the two modules are endowed with anti-oncogenic properties and contribute to the anti-tumor activity of ATRA and/or Lapatinib. To address the point with a direct approach, we selected representative miRs from *Module-1* (miR-29a-3p; miR-874-3p) and *Module-3* (miR-575; miR-1225-5p) and we performed functional studies following transient transfection of miR-mimics (Suppl. Fig. S3A).

**Figure 6:**
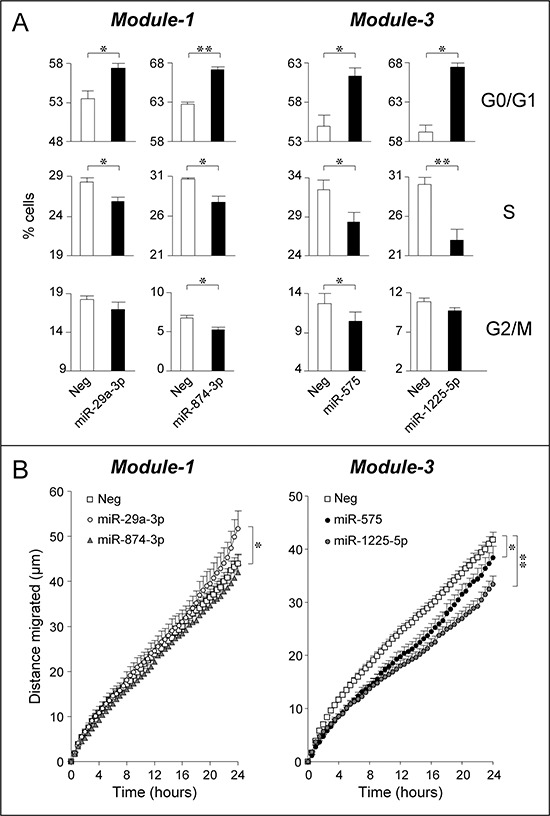
Over-expression of selected *Module-1* and *Module-3* miRs up-regulated by ATRA and/or Lapatinib: effects on SKBR3 cell-cycle and motility *SKBR3* cells were co-transfected with the indicated miRs (30 nM) or an appropriate control miR (Neg) and the pEGFP-N1 plasmid (0.01 nM) allowing expression of the GFP protein. **A.** Forty eight hours following transfection, cells were collected, fixed and stained with propidium iodide before subjecting them to FACS analysis. The bar graphs show the percentage of GFP-positive cells transiting through the indicated phase of the cell-cycle. Each value is the mean ± SD of three replicate cultures. *Significantly different (*p* < 0.05, Student's *t*-test). **Significantly different (*p* < 0.01, Student's *t*-test). **B.** Forty eight hours after transfection with the indicated miRs, *SKBR3* cells were seeded in 24-well plates and random cell motility of GFP-positive cells was evaluated by time-lapse microscopy. Each value is the mean ± SE of at least 38 individual cells. *Significantly different (*p* < 0.05, two-way ANOVA Bonferroni post-test). **Significantly different (*p* < 0.01, two-way ANOVA Bonferroni post-test).

The effect of mir-29a-3p, miR-874-3p, miR-575 and miR-1225-5p on cell growth and survival was evaluated following separate over-expression of the single miRs in exponentially growing *SKBR3* cells (Fig. 6A). The action of the four miRs on the cell-cycle are consistent with a growth inhibitory response. Indeed, all the miRs increase the percentage of cells transiting through the G0/G1-phase at the expense of the S-phase. In the case of miR-874-3p and miR-575, a concomitant reduction in the G2/M-phase is observed. The growth inhibitory action of miR-29a-3p, miR-874-3p, miR-575 and miR-1225-5p over-expression is not accompanied by apoptotic responses, as none of the miRs considered affects the basal levels of apoptosis-associated caspase-3/7 enzymatic activity (data not shown). The proposed anti-proliferative action of the selected *Module-1* and *Module-3* miRs is consistent with the predicted anti-oncogenic properties. In addition, induction of these miRs is likely to contribute to the anti-proliferative responses triggered by ATRA and/or Lapatinib in *SKBR3* cells.

The *General* and/or *Impact Scores* calculated in the small GSE38867 breast cancer dataset indicate the miR expression profiles of *Module-1* and, to a lesser extent, *Module-3* show a higher degree of similarity in NT than in DCIS, IDC or MET. The finding suggests a possible role of these miRs in regulating the invasive and metastatic behavior of breast cancer cells which is suppressed by ATRA and/or Lapatinib (see Fig. 1C). Thus, miR-29a-3p, miR-874-3p, miR-575 and miR-1225-5p were tested for their action on *SKBR3* cell random-motility by time-lapse microscopy (Fig. 6B). With regards to *Module-1*, over-expression of miR-874-3p does not alter cell motility, while over-expression of miR-29a-3p increases cell-migration. This last finding is consistent with a positive action on EMT [42], a major determinant of cell motility. In contrast, over-expression of *Module-3* miR-575 and miR-1225-5p inhibits *SKBR3* random motility. Our results indicate that miR-575 and miR-1225-5p up-regulation contributes to the anti-motility action of ATRA and/or Lapatinib. By converse, up-regulation of miR-29a-3p plays a detrimental role in the process. The data support the involvement of *Module-1* and *Module-3* miRs in breast cancer invasiveness.

The effects on two important processes associated with tumor progression are consistent with the idea that the tested miRs are endowed with anti-oncogenic properties.

### Functional validation studies with representative *Module-2* miRs in SKBR3 cells

We performed functional validation studies with selected *Module-2* miRs. The experiments were aimed at testing the oncogenic properties of *Module-2* miRs and the role played by their down-regulation in the anti-tumor action of ATRA and/or Lapatinib. For these studies, we selected miR-125a-5p, miR-193a-5p as well as miR-210-3p and we evaluated the effects of miR over-expression (Suppl. Fig. S3B) on the growth, apoptosis and motility of *SKBR3* cells.

**Figure 7:**
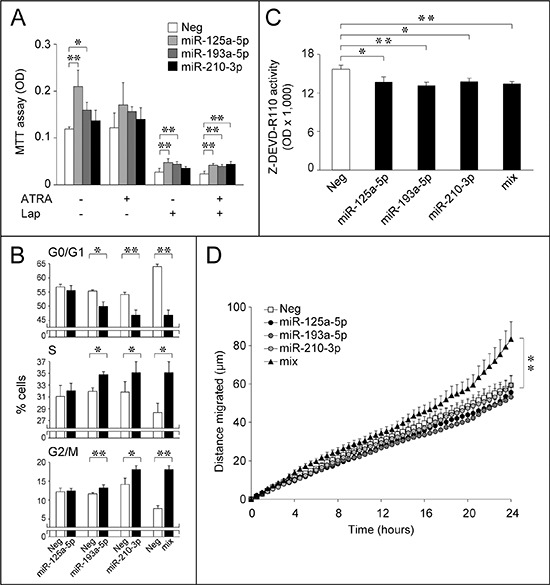
Over-expression of selected *Module-2* miRs down-regulated by ATRA and/or Lapatinib: effects on SKBR3 growth, cell-cycle, apoptosis and motility **A.** *SKBR3* cells were transfected with the indicated miRs or an appropriate control miR (Neg). Twenty four hours following transfection, cells were challenged with vehicle (DMSO), ATRA (100 nM), Lapatinib (Lap, 100 nM) or the combination of the two compounds for a further 48 hours. Cells were subject to an MTT assay for the quantitative determination of cell growth. Each value is the mean ± SD of 5 separate cultures. *Significantly different (*p* < 0.05, Student's *t*-test). **Significantly different (*p* < 0.01, Student's *t*-test). **B.** Logarithmically growing *SKBR3* cells were co-transfected with the indicated miRs (30 nM) alone or in combination (10 nM each; mix) and the pEGFP-N1 plasmid (0.01 nM) allowing expression of the GFP protein. The bar graphs show the percentage of GFP-positive cells transiting through the indicated phase of the cell-cycle. Each value is the mean ± SD of three replicate cultures. *Significantly different (*p* < 0.05, Student's *t*-test). **Significantly different (*p* < 0.01, Student's *t*-test). **C.** *SKBR3* cells were transfected with the indicated three miRs alone or in combination (10 nM each; mix) and the negative control miR (Neg). Forty eight hours after transfection, cells were lysed and caspase 3/7 enzymatic activity was determined. The values represent the mean + SD of three replicate cultures. *Significantly different (*p* < 0.05, Student's *t*-test). **Significantly different (*p* < 0.01, Student's *t*-test). **D.** Forty eight hours after transfection with the indicated 3 miRs alone or in combination (10 nM each; mix) and the negative control miR (Neg) in the presence of pEGFP-N1 as in (B), *SKBR3* cells were seeded in 24-well plates and random cell motility of individual GFP-positive cells was evaluated by time-lapse microscopy. Each value is the mean ± SE of at least 27 individual cells. **Significantly different (*p* < 0.01, two-way ANOVA Bonferroni post-test).

The significance of the selected miRs for the growth-inhibitory process was established in basal conditions, and following treatment with ATRA and Lapatinib alone or in combination (Fig. 7A). Forced expression of miR-125a-5p and miR-193a-5p increases the basal growth of *SKBR3* cells. A similar trend is observed following 48 hours of treatment with a sub-optimal concentration of ATRA, which causes no significant growth-inhibition in our experimental conditions. MiR-125a-5p and miR-193a-5p partially reverse the anti-proliferative effects of Lapatinib, while all the miRs considered, including miR-210-3p, contrast growth-inhibition by ATRA+Lapatinib. Thus, down-regulation of these *Module-2* miRs by ATRA and/or Lapatinib may contribute to the observed anti-proliferative/cyto-toxic activity. In the case of miR-193a-5p and miR-210-3p, growth stimulation is probably the result of cell-cycle regulation, as these miRs decrease the number of cells transiting through the G0/G1-phase and increase the S and G2/M phase cell population (Fig. 7B). Interestingly, the modulatory effects on the cell-cycle are enhanced when cells are transfected with a mixture of the three miRs, suggesting a coordinated action. The effects of the three selected miRs extend to cell survival. Indeed, miR-125a-5p, miR-193a-5p and miR-210-3p over-expression diminishes caspase-3/7 activity, indicating an anti-apoptotic effect (Fig. 7C). *SKBR3* cell-survival is not further augmented by combined transfection of the three miRs, suggesting common underlying mechanisms. This supports the idea that down-regulation of miR-125a-5p, miR-193a-5p and miR-210-3p contributes not only to the growth-inhibitory, but also to the cyto-toxic activity of ATRA and/or Lapatinib. Individual transfection of miR-125a-5p, miR-193a-5p or miR-210-3p does not alter *SKBR3* cell motility. However, combined transfection of the three miRs significantly stimulates this process (Fig. 7D). Thus, coordinated down-regulation of these miRs may contribute to the anti-motility action of ATRA and/or Lapatinib.

### Expression profiles and functional effects of *Module-2* miRs in other breast cancer cell lines

We evaluated whether the anti-oncogenic properties of *Module-2* miRs extend beyond the specific model of the *SKBR3* cells. To this purpose, we performed expression and functional studies in four cell lines representative of breast cancer heterogeneity. The panel consists of two luminal cell types characterized by *ER*-positivity (*MCF-7*) and *ERBB2* amplification (*MDA-MB-453*), as well as two *Basal/TN* cell types (*MDA-MB231* and *MDA-MB157*). The four cell lines show variable sensitivity to ATRA and Lapatinib (Suppl. Table S4).

**Figure 8:**
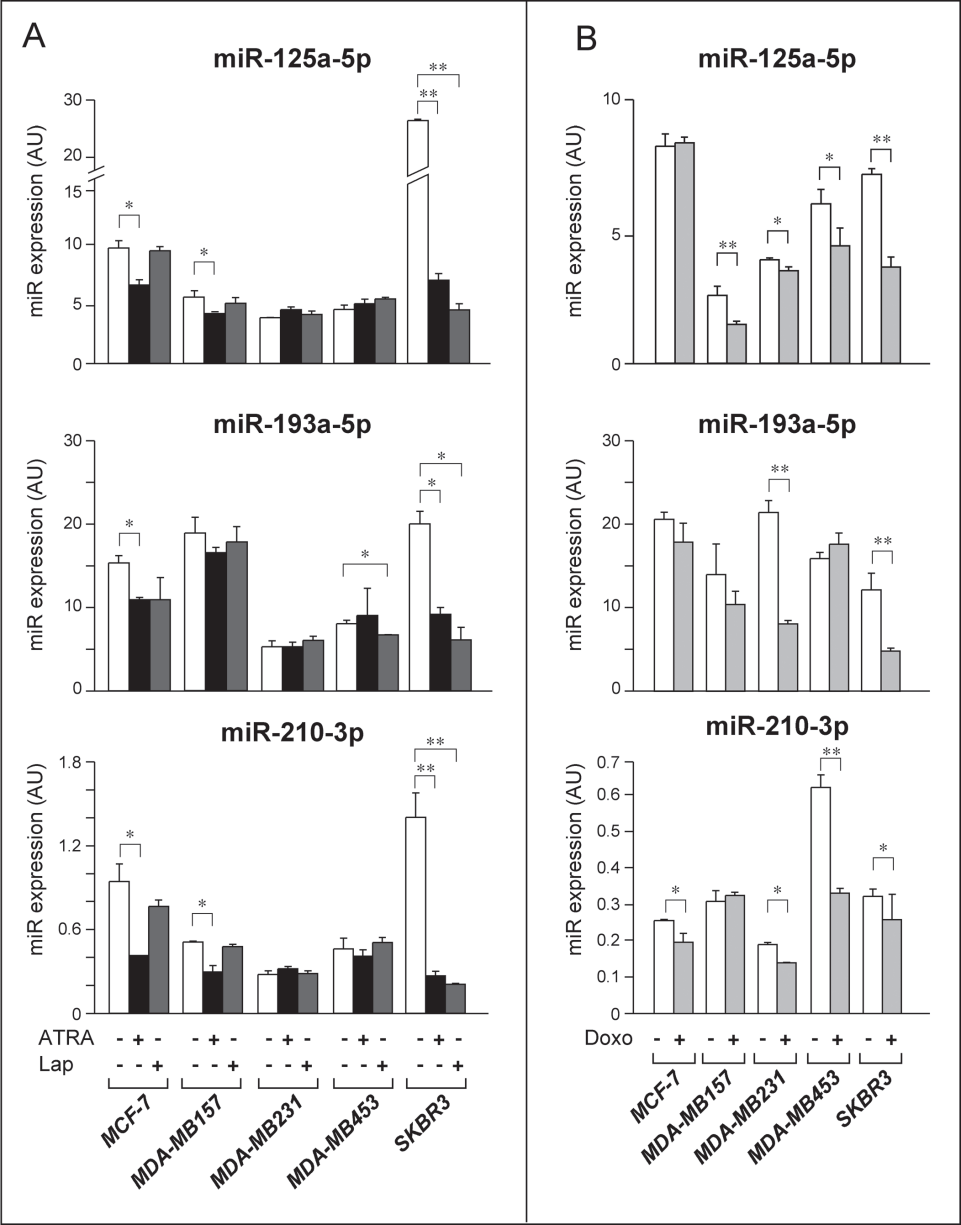
Expression of selected *Module-2* miRs in breast cancer cell lines challenged with ATRA, Lapatinib and Doxorubicin **A.** The indicated cell lines were treated with vehicle (DMSO), ATRA (*MCF-7* = 1 μM; *MDA-MB157* = 1 μM; *MDA-MB231* = 1 μM; *MDA-MB453* = 1 μM; *SKBR3* = 0.1 μM) or Lapatinib (*MCF-7* = 0.1 μM; *MDA-MB157* = 0.1 μM; *MDA-MB231* = 0.1 μM; *MDA-MB453* = 1 μM; *SKBR3* = 0.1 μM) for 30 hours. The graphs illustrate the expression profiles of the indicated *Module-2* miRs, as determined by quantitative real-time PCR (qPCR). Each qPCR value is the mean of 3 replicates ± SD. **B.** The indicated cell lines were treated with vehicle (DMSO) or Doxorubicin (*MCF-7* = 0.25 μM; *MDA-MB157* = 0.1 μM; *MDA-MB231* = 0.1 μM; *MDA-MB453* = 0.5 μM; *SKBR3* = 0.1 μM) for 24 hours. The results were obtained by qPCR analysis as in A. Each result is the mean of 3 replicates ± SD. **Significantly different (*p* < 0.01, Student's *t*-test). *Significantly different (*p* < 0.05, Student's *t*-test). Lap = Lapatinib; Doxo = Doxorubicin.

In a first set of experiments, we determined the action of ATRA and Lapatinib on the levels of miR-125a-5p, miR-193a-5p and miR-210-3p, in the four cell lines and *SKBR3* as a control. Similar to what is observed in the *SKBR3* model, ATRA down-regulates all the *Module-2* miRs considered in *Luminal*/*ER^+^ MCF-7* cells (Fig. 8A). Significant ATRA-dependent down-regulation is observed for miR-125a-5p and miR-210-3p in the other retinoid-sensitive *MDA-MB-157* cell line. In contrast, none of the miRs is regulated by ATRA in retinoid refractory *MDA-MB-231* and *MDA-MB-453* cells. The results suggest that down-regulation of miR-125a-5p, miR-210-3p and possibly miR-193a-5p is associated with the anti-proliferative action of ATRA. As for Lapatinib, the kinase inhibitor down-regulates miR-193a-5p in the *Her2^+^ MDA-MB-453* cell line. At variance with *SKBR3* cells, Lapatinib does not alter the expression of miR-125a-5p or miR-210-3p in *MDA-MB-453* cells. None of the *Module-2* miRs is regulated by the tyrosine kinase inhibitor in *Her2^−^* and Lapatinib-refractory *MCF-7*, *MDA-MB157* and *MDA-MB231*.

We further investigated whether down-regulation of *Module-2* miRs by ATRA and/or Lapatinib is a consequence of cell growth inhibition or cytotoxicity and it is independent of the anti-tumor agent considered. To this purpose, we evaluated the expression of miR-125a-5p, miR-193a-5p and miR-210-3p following treatment with doxorubicin (Doxo), an anti-tumor agent inhibiting the growth of all our cell lines in the concentration range considered (Fig. 8B). At concentrations reducing cell numbers by approximately 25% in 24 hours (Suppl. Table S4), miR-125-5p is significantly down-regulated by Doxo in *MDA-MB-157*, *MDA-MB-231*, *MDA-MB453* and *SKBR3* cells. Doxo causes a significant down-regulation of miR-193a-5p in *MDA-MB231* and *SKBR3* cells. Except for *MDA-MB157*, Doxo reduces the expression of miR-210-3p in all the cell lines tested. Taken together, the data obtained support the idea that down-regulation of *Module-2* miRs is a general response of all breast cancer cell lines to growth inhibitory or cyto-toxic stimuli.

**Figure 9:**
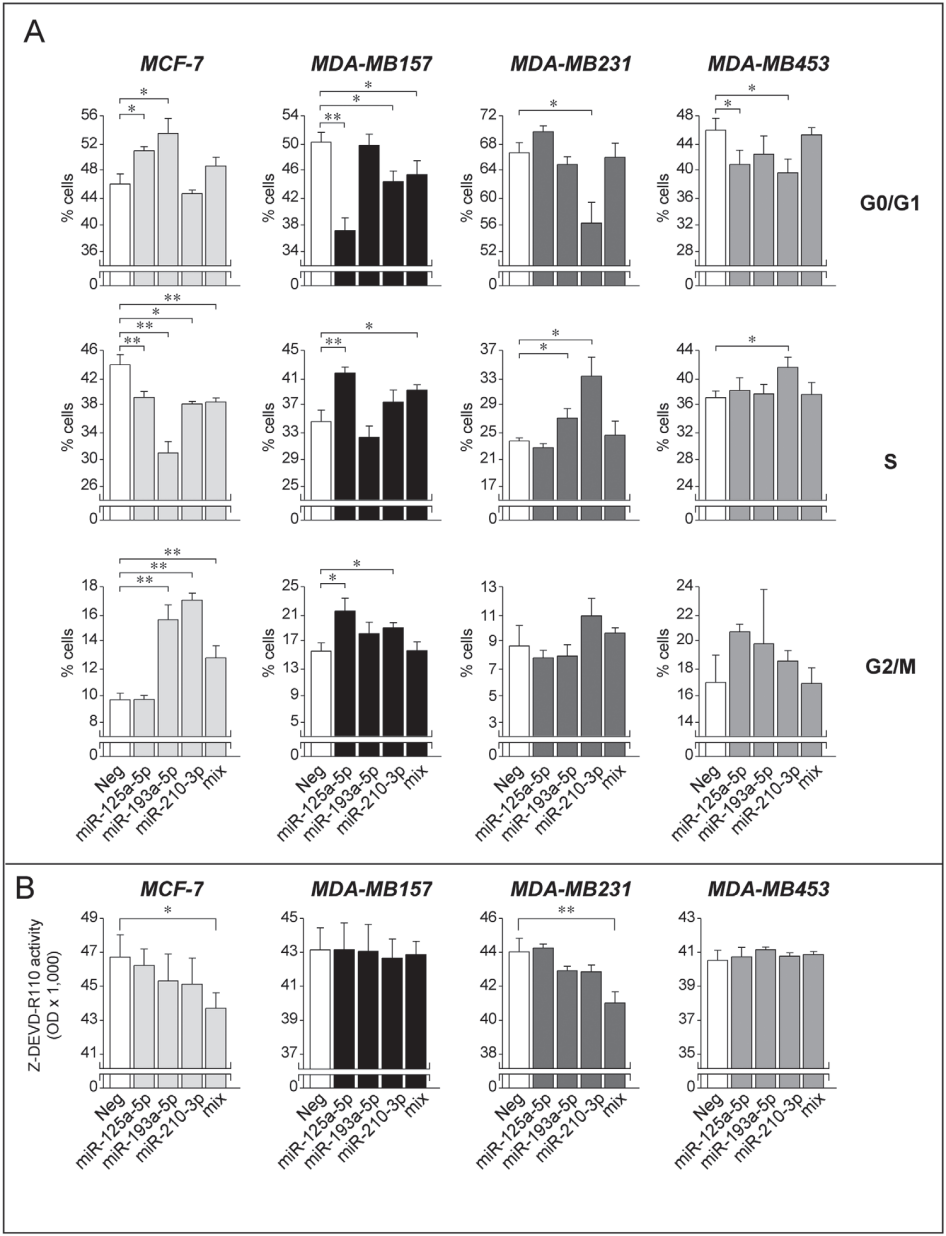
Over-expression of selected *Module-2* miRs; effects on cell-cycle and apoptosis in breast cancer cell lines **A.** The listed cell lines were co-transfected with the indicated miRs (30 nM) alone or in combination (10 nM each; mix) and the pEGFP-N1 plasmid (0.01 nM) allowing expression of the GFP protein. The bar graphs show the percentage of GFP-positive cells transiting through the indicated phase of the cell-cycle. Each value is the mean ± SD of 3 replicate cultures. *Significantly different (*p* < 0.05, Student's *t*-test). **Significantly different (*p* < 0.01, Student's *t*-test). **B.** The indicated cell lines were transfected with miR-125a-5p, miR-193a-5p and miR-210-3p and the negative control miR (Neg). Forty eight hours after transfection, cells were lysed and caspase 3/7 enzymatic activity was determined. The values represent the mean ± SD of three replicate cultures. *Significantly different (*p* < 0.05, Student's *t*-test). **Significantly different (*p* < 0.01, Student's *t*-test).

In a series of functional studies, we evaluated whether the growth stimulatory, anti-apoptotic and pro-motility actions of *Module-2* miR-125a-5p, miR-193a-5p and miR-210-3p are limited to *SKBR3* or they extend to the other breast cancer cell lines considered. The effects on cell growth were determined by measuring perturbations in the cell-cycle following over-expression of the three *Module-2* miRs alone or in combination. Over-expression of the three miRs in all the cell lines following transfection was confirmed by qPCR (data not shown). In *MDA-MB157* cells, forced expression of miR-125a-5p and miR-210-3p replicates the proliferative effect established in the *SKBR3* counterparts. In fact, the two miRs contract the G0/G1 phase and expand the S-phase of the cell cycle (Fig. 9A). The growth stimulatory action is maintained, if the three miRs are simultaneously transfected, which is suggestive of cooperative effects. Similar pro-growth responses are observed following individual transfection of miR-193a-5p and miR-210-3p in *MDA-MB231* cells. In the *MDA-MB453* line, forced expression of miR 125a-5p and miR210-3p reduces the fraction of cells transiting through the G0/G1 phase, once again consistent with a proliferative action. Noticeably, individual and combined over-expression of miR-125a-5p, miR-193a-5p and miR-210-3p in the *Luminal*/*ER^+^ MCF-7* cell line exerts opposite actions which are consistent with an overall growth inhibitory effect. Indeed, individual over-expression of the three *Module-2* miRs in *MCF-7* cells results in a consistent expansion of the G0/G1 phase and/or a reduction of the S phase. These effects are accompanied by variable perturbations of the G2/M phase. Interestingly, the anti-proliferative action of miR-125a-5p in *MCF-7* cells confirms results obtained in the same cellular context by Guo *et al*. [43]. Taken together these studies indicate that the action of *Module-2* miRs on the cell-cycle is highly dependent on the cellular context considered. Nevertheless, with the exception of *MCF-7*, the three *Module-2* miRs exert a predominant growth-stimulatory effect in breast cancer cells.

As far as apoptosis is concerned, individual over-expression of miR-125a-5p, miR-193a-5p and miR-210-3p does not exert any significant effect on caspase-3/7 activation in *MCF-7*, a known caspase-3 defective cell line [43], *MDA-MB157*, *MDA-MB231* and *MDA-MB-453* cells (Fig. 9B). This is different from what is observed in *SKBR3* cells, where these miRs are characterized by an anti-apoptotic action (see Fig. 7C). However, combined transfection of the three miRs in *MCF-7* and *MDA-MB231* cells causes a significant inhibition of caspase-3/7 activity. The data support the idea that the coordinated action of *Module-2* miRs triggers not only a growth inhibitory, but also an anti-apoptotic effect in specific cellular contexts.

**Figure 10:**
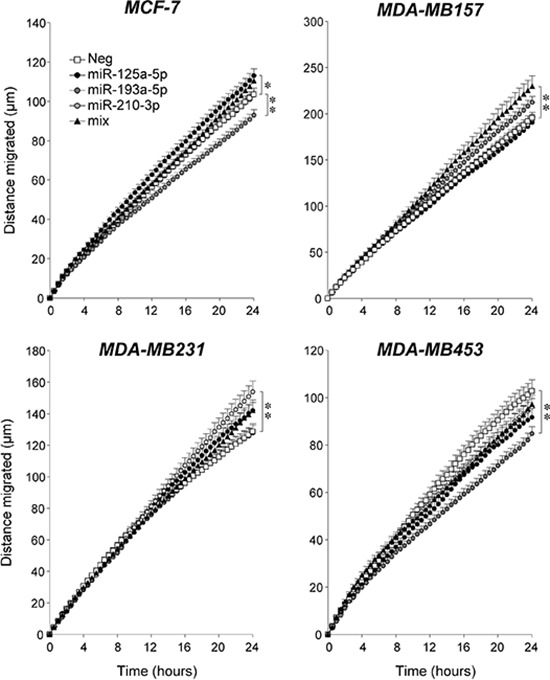
Over-expression of selected *Module-2* miRs; effects on the motility breast cancer cell lines The indicated cell lines were transfected with the indicated miRs alone (30 nM) or in combination (10 nM each; mix) and the negative control miR (Neg) in the presence of pEGFP-N1 (0.01 nM). Forty eight hours after transfection, cells were seeded in 24-well plates and random cell motility of individual GFP-positive cells was evaluated by time-lapse microscopy. Each value is the mean ± SE of at least 27 individual cells. *Significantly different (*p* < 0.05, two-way ANOVA Bonferroni post-test) **Significantly different (*p* < 0.01, two-way ANOVA Bonferroni post-test).

The effect exerted by miR-125a-5p, miR-193a-5p and miR-210-3p on the migratory behavior of breast cancer cells was investigated. Similar to what is observed in *SKBR3*, only combined transfection of the three *Module-2* miRs results in increased migration of the *Basal*/*TN* and highly motile *MDA-MB157* cells (Fig. 10). In the other *Basal*/*TN MDA-MB-231* cell line, transfection of miR-210-3p significantly increases random-motility, although a similar effect seems to occur also in the case of miR-125a-5p. In *MDA-MB-231* cells, combined transfection of the three *Module-2* miRs is no more effective than miR-210-3p. In the *Luminal MCF-7* and *MDA-MB-453* cell lines, which are characterized by much lower motility than the two *Basal*/*TN* counterparts, the picture is complex. In *MCF-7* cells, motility is increased by miR-125a-5p, it is decreased by miR-193a-5p and it is left unaffected by miR-210-3p. In *MDA-MB-453* cells, the only miR exerting an effect is miR-193a-5p, which reduces motility. Thus, the *Luminal* and *Basal/TN* phenotypes seem to represent primary determinants of the motility responses triggered by the *Module-2* miRs considered.

### Two novel targets of *Module-2* and *Module-3* miRs: HIPK2 and PLCXD1

We functionally validated if any of the predicted target-genes contained in the identified modules are indeed regulated by the corresponding miRs. For these experiments, we selected one gene from *Module-2* (HIPK2, homeodomain-interacting-protein-kinase-2) and another gene from *Module-3* (PLCXD1, phosphatidylinositol-specific phospholipase-C-X-domain-containing-1), representing potential targets of miRs which are regulated in *SKBR3* cells treated with ATRA and/or Lapatinib. In the case of both HIPK2 and PLCXD1, selection was dictated by high basal expression levels and robust fold-change upon treatment with ATRA and/or Lapatinib. HIPK2 is one of the most inter-connected targets of *Module-2* miRs and it is considered to be an onco-suppressor, as it is a pro-apoptotic agent and it inhibits the invasiveness of breast cancer cells [44–46]. The function of PLCXD1 is largely unknown although its over-expression suppresses melanoma cell growth, suggesting anti-oncogenic properties [47].

**Figure 11:**
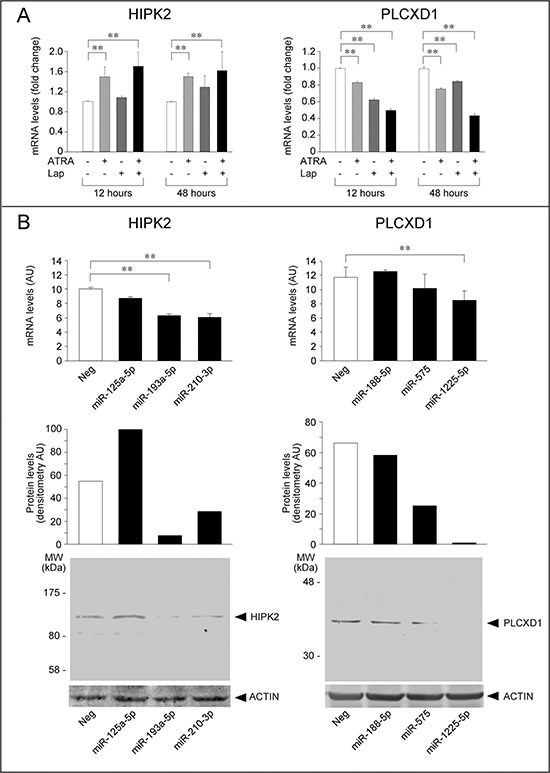
Regulation of HIPK2 and PLCXD1, two novel targets of specific miRs up- and down-regulated by ATRA and/or Lapatinib **A.** The expression pattern of HIPK2 and PLCXD1 transcripts in *SKBR3* cells challenged with ATRA (100 nM), Lapatinib (100 nM) and the combination of the two compounds for 12 and 48 hours is illustrated. The results were obtained from the gene-expression microarray experiments. Each value is expressed in fold-change relative to the vehicle treated control and it is the mean ± SD of 5 replicate arrays. **Significantly different (*p* < 0.01, Student's *t*-test). **B.** *SKBR3* cells were transfected with the indicated miRs and an appropriate control miR (Neg). After 48 hours, total RNA was extracted and the levels of HIPK2 and PLCXD1 mRNAs were determined with the use of specific Taqman assays (upper graphs). **Significantly different (*p* < 0.01, Student's *t*-test). In the same experimental conditions, the levels of HIPK2 and PLCXD1 proteins were determined by Western blot analysis (bottom images). The filters used for Western blot analyses were re-blotted with an anti-β-actin antibody, which demonstrates that the same amount of protein was loaded in each lane. Densitometric quantification of the HIPK2 and PLCXD1 bands is shown by the middle bar graphs.

In *SKBR3* cells, the HIPK2 mRNA is rapidly up-regulated by ATRA (Fig. 11A). Up-regulation is observed also with Lapatinib, although the effect is more delayed. The increase in HIPK2 mRNA is higher in cells exposed to ATRA+Lapatinib relative to ATRA alone. Over-expression of miR-193a-5p or miR-210-3p (*Module-2*) in *SKBR3* cells down-regulates the HIPK2 transcript (Fig. 11B). In contrast, transfection of miR-125a-5p has no significant effect on the steady-state levels of the mRNA. Down-regulation of the HIPK2 mRNA by miR-193a-5p and miR-210-3p translates into down-regulation of the corresponding protein. Thus, HIPK2 is a novel and common target of miR-193a-5p and miR-210-3p. In *SKBR3* cells, down-regulation of the two miRs by ATRA and ATRA+Lapatinib is likely to contribute to HIPK2 up-regulation by the two stimuli.

The PLCXD1 transcript is down-regulated by ATRA and Lapatinib at both 12 and 48 hours. Down-regulation is enhanced by the combination of ATRA+Lapatinib at the two time points considered (Fig. 11A). Forced expression of miR-575 and miR-1225-5p (*Module-3*) decreases the levels of the PLCXD1 mRNA, although the effect reaches statistical significance only in the case of miR-1225-5p (Fig. 11B). The two miRs also cause a reproducible and robust reduction in the amounts of the PLCXD1 protein. Thus, the regulatory effect exerted by miR-575 is predominantly due to inhibition of protein synthesis, while miR-1225-5p affects both RNA stability and translation efficiency. A third miR belonging to *Module-3*, i.e. miR-188-5p, does not alter the levels of either the PLCXD1 mRNA or protein. The data indicate that PLCDX1 is a novel and common target of miR-575 and miR-1225-5p. In addition, they support the idea that up-regulation of the two miRs by ATRA, Lapatinib and ATRA+Lapatinib contributes to the down-regulation of PLCXD1 in *SKBR3* cells.

## DISCUSSION

*SKBR3* cells are characterized by co-amplification of the genes coding for HER2 and the nuclear retinoic acid receptor, RARα, and they are representative of a novel subgroup of HER2^+^ breast tumors with selective sensitivity to simultaneous targeting of RARα with ATRA and HER2 with Lapatinib [1]. In this study, the *SKBR3* breast cancer model is used to demonstrate that ATRA and Lapatinib regulate 174 miRs and predicted target-mRNAs which modulate proliferation, survival and invasiveness, contributing to the anti-tumor action of the two compounds. About one quarter of ATRA and/or Lapatinib regulated miRs (47/174) and potential target-mRNAs are organized in four highly interconnected modules.

*Module-1* consists of miRs whose expression is increased by Lapatinib and enhanced by ATRA+Lapatinib. In *Module-1*, up-regulation of miR-26a, mir-29a-3p, miR-30d, miR-134, miR-181a, miR-320c, miR-874-3p, miR-1226, miR-1181 and miR-1915 by Lapatinib and/or ATRA is entirely consistent with the generally accepted anti-oncogenic action of these miRs in breast cancer or other tumors (Suppl. Table S5). In fact, miR-26a is reported to inhibit the growth and motility of breast cancer cells [48]. The expression profile of miR-134 in the same tumor type suggests that the miR is an onco-suppressor [49]. Interestingly, miR-134 targets HER2 [50], whose gene is amplified and active in *SKBR3* cells [1]. MiR-181a is also likely to be an onco-suppressor, as it reduces the ability of breast cancer cells to induce mammospheres [51]. The only two miRs whose up-regulation by Lapatinib and ATRA+Lapatinib is against the predicted anti-oncogenic action are miR-150, which promotes the growth of certain mammary carcinoma cells [52], and miR-762, which is up-regulated in oral squamous carcinomas [53]. With the use of an over-expression approach, two of the miRs belonging to *Module-1*, i.e. mir-29a-3p and miR-874-3p, were directly evaluated for their action on *SKBR3* cell survival, growth and motility. Forced expression of neither mir-29a-3p nor miR-874-3p affects the process of apoptosis, indicating that the two miRs are not involved in the regulation of *SKBR3* cell survival. In contrast, mir-29a-3p and miR-874-3p play a role in the process of cell proliferation. In particular, the anti-proliferative effect of mir-29a-3p recapitulates what was observed in two other breast cancer cell lines [54], while growth inhibition by miR-874-3p is consistent with its tumor-suppressor role in non-small-cell lung as well as head and neck cancers [55–57]. As for this last miR, it would be interesting to know whether miR-874-3p exerts the same suppression of the cancer stem-cell phenotype described in lung cancer also in mammary tumors. Unlike miR-874-3p, miR-29a-3p over-expression exerts a significant stimulatory effect on *SKBR3* cell motility. This last observation is unexpected, as the miR has been reported to down-regulate Sparc, a protein stimulating breast cancer cell invasiveness [58].

In *Module-2*, miR down-regulation is predominantly due to ATRA and ATRA+Lapatinib. Down-regulation of miR-96, miR-203 and miR-210-3p is consistent with the predicted oncogenic properties of the three miRs [59–64]. Noticeably, miR-96 promotes breast cancer cell proliferation targeting FOXO3A, which is a major node of ATRA/Lapatinib cross-talk in *SKBR3* cells [1]. MiR-203 is under-expressed in invasive relative to non-invasive breast cancer cells [65], although the miR seems to function as a tumor-suppressor in triple-negative carcinomas [66]. In contrast, down-regulation of miR-15b, miR-149 and miR-183 is hard to reconcile with their predicted anti-oncogenic potential [67–70]. With the same over-expression approach used for *Module-1*, we evaluated the action of miR-125a-5p, miR-193a-5p and miR-210-3p on the proliferation, survival and motility of *SKBR3* cells. The results indicate that individual down-regulation of the three miRs contributes to the anti-proliferative and anti-apoptotic responses triggered by ATRA and/or Lapatinib, as miR over-expression stimulates *SKBR3* cell growth and survival. As for the proliferative action of miR-193a-5p and miR-210-3p, this is the result of a small, albeit consistent and significant, effect favoring exit of *SKBR3* cells from the G0/G1-phase of the cell cycle. In the case of miR-193a-5p, it is interesting that the miR has been reported to inhibit HER2 expression [50]. Thus down-regulation of miR-193a-5p by ATRA is against an involvement in the inhibitory effect exerted by the retinoid on HER2 expression in *SKBR3* cells [1]. The data on cell motility indicate that the action of miR-125a-5p, miR-193a-5p and miR-210-3p needs to be coordinated to obtain a pro-motility response. All this demonstrates that *Module-2* miRs play a role in the control of *SKBR3* cell cycle and motility, which is in accordance with our process enrichment analysis indicating involvement of miR target-mRNAs in EMT and regulation of growth factors involved in G1- to S-phase transition (see Table 2).

*Module-3* contains miRs which are predominantly up-regulated by ATRA and ATRA+Lapatinib. Up-regulation of miR-21, miR-22, miR-103, mir-141 and miR-200c supports the anti-oncogenic properties of these regulatory RNAs (Suppl. Table S5). Specifically, miR-22 induces senescence [71] and inhibits invasiveness [72] of breast cancer cells. MiR-103 inhibits mammary tumor stem-cell growth [67]. Mir-141 and miR-200c belong to the same family of miRs and they inhibit the process of EMT in breast cancer cells [20, 73]. Although miR-21 is generally considered to be oncogenic, we recently demonstrated that it is induced by ATRA in estrogen-receptor-positive mammary tumor cells. In this cellular context, miR-21 counteracts the growth-inhibitory action, while it contributes to the anti-motility effects of ATRA [27]. As for the potential anti-oncogenic miRs belonging to *Module-3*, we focused our attention on miR-575 and miR-1225-5p, determining the functional activity of these miRs in *SKBR3* cells. Similar to what is observed in the case of the two miRs belonging to *Module-1*, both miR-575 and miR-1225-5p exert an anti-proliferative action which involves effects on the cell-cycle. The two miRs also inhibit *SKBR3* cell random-motility, which is supportive of an anti-metastatic action. Thus, miR-575 and miR-1225-5p may represent *bona fide* onco-suppressors in *Her2*^+^ breast cancer [74, 75].

The expression pattern of the five miRs (miR-148a; miR-193a-3p; miR-205; miR-375; miR-660) present in *Module-4* is complicated, as the overall effect of the ATRA+Lapatinib combination is the result of an opposite regulatory action exerted by ATRA and Lapatinib alone. For this reason, no functional studies were performed with any of the *Module-4* miRs. Given the complex expression pattern of miR-205, miR-375, miR-660, miR-193a-3p and miR-148a, it is difficult to discuss the relevance of the single members of this module for the growth and progression of breast cancer. Nevertheless, high levels of miR-375 are present in luminal mammary tumors and the miR seems to inhibit EMT [76]. Thus overall down-regulation of miR-375 by ATRA+Lapatinib may be detrimental for the anti-metastatic activity of this drug combination. In addition, pathway enrichment analysis indicates that the genes regulated by *Module-4* miRs control two specific aspects of cancer cell biology, which are predominantly related to motility and invasiveness.

Identification of these four sets of miRs is of more general interest for breast cancer biology and their relevance goes beyond the involvement in ATRA and/or Lapatinib anti-tumor action. The *Whole* miR fingerprint defined by all the miRs contained in *Module-1* to *Module-4* is associated with the progression of breast carcinoma independently of their *HER2* status, as indicated by studies comparing the miR expression profiles in a small cohort of cases consisting of normal mammary gland tissue and matched DCIS, IDC and MET samples. In fact, two calculated similarity scores based on all miRs (*General Score*) and the subset of over-connected miRs (*Impact Score*) are higher in normal mammary tissue than in patient-matched DCIS, IDC and MET. This indicates that treatment of the neoplastic *SKBR3* cells with ATRA and/or Lapatinib changes the miR expression profile, making it more similar to that observed in normal mammary gland cells. The *General Score* and the *Impact Score* calculated for *Module-1*, *Module-2* and, to a lesser extent, *Module-3*, separately, also show a trend towards a decrease in similarity score with disease progression. These observations are consistent with up-regulation of *Module-1* and *Module-3* and down-regulation of *Module-2* miRs by an effective anti-proliferative, cyto-differentiating and apoptotic combination of drugs. The data showing an association between our overall miR fingerprint are supported by the *Impact Scores* calculated in the much larger TCGA dataset of breast cancer samples. In this dataset the highest similarity scores are observed in cases characterized by a low staging and size index, and there is a progressive diminution in the scores as staging and size increase. The same associations are determined if the fingerprint of over-connected miRs belonging to *Module-2* only is considered. Interestingly, the majority of genes whose expression is controlled by *Module-2* miRs are involved in the regulation of G1- to S-phase progression and EMT. Thus, it is relevant that the *Module-2 Impact Score* is inversely correlated with the proliferation score of the TCGA tumor samples. Regulation of EMT is of particular significance for the inverse association with breast cancer progression, as the process is crucial for the homeostasis of cancer stem-cells and the invasive/metastatic behavior of neoplastic cells [36, 77]. Taken together the results obtained indicate that the overall miR fingerprint and the smaller *Module-2* fingerprint have diagnostic and prognostic potential.

Given the proposed pro-oncogenic properties and the particular relevance of *Module-2* miRs for the growth and progression of breast cancer which is suggested by the associations observed in the clinical dataset, we focused our attention on this specific module. We investigated whether the regulation of miR-125a-5p, miR-193a-5p and miR-210-3p by ATRA, Lapatinib and Doxo as well as their activity on growth, survival and motility is limited to *SKBR3* cells or extends to other cellular models representative of breast cancer heterogeneity. For these studies, we selected four cell lines [78] characterized by distinct phenotypes and different responsiveness to ATRA and Lapatinib. In general, the expression results are consistent with the idea that the three miRs are endowed with pro-oncogenic properties in *Her2^+^* (*SKBR3*; *MDA-MB453*), *Basal*/*TN* (*MDA-MB157*; *MDA-MB231*) and *Luminal*/*ER^+^* (*MCF-7*) breast cancer cells. In fact, the three miRs are down-regulated by Doxo in almost all the cell lines tested and by ATRA or Lapatinib in the counterparts sensitive to the anti-proliferative action of the two compounds.

The general oncogenic properties of *Module-2* miRs are confirmed by the effects of miR-125a-5p, miR-193a-5p and miR-210-3p over-expression on the growth of *MDA-MB157*, *MDA-MB231* and *MDA-MB453* which are largely in line with what is observed in *SKBR3* cells. The only exception is represented by *MCF-7* cells which show complex cell-cycle responses. In fact, the expansion of the G0/G1 phase or the contraction of the S phase observed upon miR-125a-5p, miR-193a-5p and miR-210-3p over-expression suggests a growth inhibitory action, although this is not accompanied by the expected effects on the G2/M phase. Interestingly, this is consistent with the function of miR-125a as an oncosupressor suggested by Guo *et al*. [43] in the *MCF-7* model. The results need to be confirmed in other *Luminal*/*ER^+^* and retinoid-sensitive cellular models, but they suggest that these *Module-2* miRs may be anti-oncogenic in this type of mammary tumor. As for cell survival, individual *Module-2* miRs seem to be devoid of significant apoptotic or anti-apoptotic activity in the cell lines considered. However, simultaneous over-expression of miR-125a-5p, miR-193a-5p and miR-210-3p reduces caspase-3/7 in *MCF-7* and *MDA-MB231* cells, similar to what is observed in the *SKBR3* model. In the case of cell motility, the effects of the three *Module-2* miRs are variable and highly dependent on the cellular context, preventing us to draw any general conclusion.

A final result of our study is the identification of HIPK2 as a novel target of miR-193a-5p and miR-210-3p (*Module-2*). In fact, over-expression of the two individual miRs in *SKBR3* cells results in down-regulation of HIPK2 mRNA and/or protein. Thus, miR-193a-5p and miR-210-3p are likely to contribute to the up-regulation of HIPK2 by ATRA and ATRA+Lapatinib in *SKBR3* cells. Up-regulation of HIPK2 in our model is consistent with the anti-oncogenic properties of this homeodomain-interacting serine/threonine protein kinase [44, 46, 79]. The kinase has been reported to promote an apoptotic response and to be a mediator of UV-dependent cytotoxicity [79]. In addition, HIPK2 has been shown to inhibit the growth and invasiveness of breast cancer cells [44, 46]. PLCXD1 is a newly identified target transcript of miR-575 and miR-1225-5p belonging to *Module-3*. Down-regulation of the protein by ATRA, Lapatinib and the combination in *SKBR3* cells is likely to be, at least partly, the consequence of miR-575 and miR-1225-5p up-regulation. Down-regulation of PLCXD1 in our experimental conditions is consistent with the idea that this phospholipase C is endowed with oncogenic properties. This is contrary to the results reported in the only study available on PLCXD1 which showed growth-suppressive effects in melanoma cells [47].

In conclusion, our study demonstrates that coordinated regulation of numerous miRs of potential diagnostic and prognostic value plays an important role in the anti-tumor action of ATRA and Lapatinib in the *SKBR3* breast carcinoma cell line. Some of these effects are of general significance, as they are replicated in other breast cancer cell lines representing the heterogeneity of this disease and with a common anti-tumor agent like Doxo. It remains to be established whether the pro- and anti-oncogenic properties of *Module-1* to *-4* miRs can be extended to tumors other than breast cancer. With respect to this, it is noticeable that members of the miR-125 family and miR-193a-3p/-5p are known to act as both oncogenes or anti-oncogenes in different types of carcinomas [80, 81].

## MATERIALS AND METHODS

### Chemicals and cells

ATRA was from Sigma (St Louis, MI, USA) and Lapatinib from LC Laboratories (Woburn, MA, USA). *SKBR3*, *MD-MB157*, *MDA-MB231*, *MCF-7* and *MDA-MB435* cells (ATCC, American-Type-Culture-Collection) were seeded at 3 × 10^5^ cells/cm^2^, cultured and treated as described [1].

### Transfections and cell viability/proliferation assays

Transfections of *SKBR3* and other breast cancer cell lines (3.5 × 10^4^ cells/cm^2^) with 30 nM miR mimics (Life Technologies, Carlsbad, CA) and 0.01 nM pEGFP-N1 plasmid (Clontech, Mountain View, CA, USA) were performed using Lipofectamine-2000 (Life Technologies). A list of the miR-mimics is present in Suppl. Table S6. Cell numbers and viability were determined with an automated Coulter Counter (BD Biosciences, Milan, Italy). Proliferation was also evaluated with the 3-(4, 5-Dimethyl-2-thiazolyl)-2, 5-diphenyl-2H-tetrazolium bromide (MTT) assay [82].

### Cell-cycle analysis and single-cell random motility assays

Cell-cycle analysis was conducted on miR-mimic and pEGFP-N1 co-transfected *SKBR3* cells as described [83]. Random-motility assays were performed on collagen/BSA coated culture wells by time-lapse microscopy [27].

### Western blots and caspase 3/7 assays

Western blot analyses were performed with anti-PLCXD1 (Abcam, Cambridge, UK; ab67147), anti-HIPK2 (Abcam, ab108543) and anti-β-actin (Santa Cruz Biotechnology, sc-1615) antibodies. The secondary antibodies were cyanine-3 or cyanine-5 conjugated anti-IgG antibodies (Ambion). Blots were analyzed using an automated fluorescence scanner (Typhoon, GE-Healthcare, Pittsburgh, PA, USA). Apoptotic assays were performed with Apo-ONE-Homogeneous-Caspase-3/7 Assay (Promega, Madison, WI, USA).

### PCR and miR microarrays

RNA was prepared using the mercury-RNA-isolation-kit (Exiqon, Copenhagen, Denmark) and it was reverse-transcribed with the GeneAmp-RNA-PCR-Core-Kit (Applied Biosystems). PCR was performed with the 7300 system (Applied Biosystems, software version 1.3.1). Mature miR levels were measured with Taqman assays (Applied Biosystems). Mature miRs were also quantified using the Exiqon-LNA-qPCR system using miR-425 as a normalising control. HIPK2 and PLCXD1 mRNAs were determined with Taqman assays (Applied Biosystems) following reverse transcription of RNA with the GeneAmp RNA PCR Core Kit (Applied Biosystems). A list of the Taqman and Exiqon assays are shown in Suppl. Table S6.

Human miR microarrays (Release 16.0, 8 × 60K, G4870A) were from Agilent (Santa Clara, CA, USA). RNA was prepared from five biological replicate cell preparations using the miRNeasy-Mini-Kit (Qiagen, Milan Italy). RNA labeling, hybridization and data extraction (Feature Extraction software v. 10.5) were performed according to Agilent protocols. The results of the miR microarray experiments were deposited in the ArrayExpress database under the accession number, E-MTAB-3097.

### Integrated miR/mRNA data analysis

Two active datasets from *SKBR3* cells were used for the analysis: 1) miRs selected for significant changes in expression by one of the factors in the described microarray experiments (ATRA, Lapatinib, or interaction, 36 h treatment; two-way ANOVA, *p* < 0.01); 2) mRNAs selected in the same way from previously obtained microarray data (E-MEXP-3192; http://www.ebi.ac.uk/arrayexpress) (48 hours; *p* < 0.001). The MAGIA (MiRNA-And-Genes-Integrated-Analysis) web-tool (http://gencomp.bio.unipd.it/magia/start/) [84] was used to predict miR/target mRNA interactions in these two datasets (Suppl. Fig. S2). The steps for the analysis were: 1) identification of putative miR/target-mRNA pairs by anyone of three prediction algorithms (PITA, miRanda, TargetScan, filters set as default); 2) for each of these pairs, selection of those showing a significant negative correlation between miR and mRNA-expression (Spearman correlation < −0.9, *q*-value < 0.05).

Cytoscape [85] was used to analyze the predicted interaction networks (Fig. 2C). We performed enrichment analysis of the target genes in each Module on Metacore-annotated process networks (http://thomsonreuters.com/metacore/).

### Analysis of miR fingerprint in clinical breast cancer samples

We used two datasets: GSE38867 from the NCBI Gene Expression Omnibus (http://www.ncbi.nlm.nih.gov/gds) and the Breast Invasive Carcinoma miRNAseq and Gene expression (RNA-seq) dataset from the Cancer Genome Atlas (TCGA, http://cancergenome.nih.gov). The similarity score was defined as follows:

$$\sum_{\text{i}\epsilon \text{n}}{\text{w}}_{\text{i}}{\text{x}}_{\text{i}}$$

where w_i_ is the weight of i^th^ miR in signature (+1 for miRs in *Module-1* and *-3*; -1 for miRs in *Module-2* and *-4*) and x_i_ is the expression value of i^th^ miR in test samples. We calculated two types of similarity scores: a *General Score* based on all the miRs and an *Impact Score* based on the subset of over-connected miRs (miRs with degree >50, i.e. those with more than 50 predicted target-mRNAs). The scores were analyzed in relation to tumor progression stages in the first dataset, and to several parameters in the second dataset: normal tissue versus tumor, tumor size and stage, tumor proliferation score [86] and overall survival. Survival analyses (Kaplan-Meier, rank [87] and Cox-proportional-hazard tests) were performed using the “survival” package in R Bioconductor (http://www.bioconductor.org).

## SUPPLEMENTAY FIGURES AND TABLES










