# Supplementary material for: MicroRNA networks regulated by all-trans retinoic acid and Lapatinib control the growth, survival and motility of breast cancer cells
Source: Oncotarget. 2015 Apr 18;6(15):13176–200. doi: 10.18632/oncotarget.3759 (PMC4537007; doi:10.18632/oncotarget.3759)
Supplement: Supplementary file 1 [file oncotarget-06-13176-s001.pdf]

SUPPLEMENTAY FIGURES AND TABLES

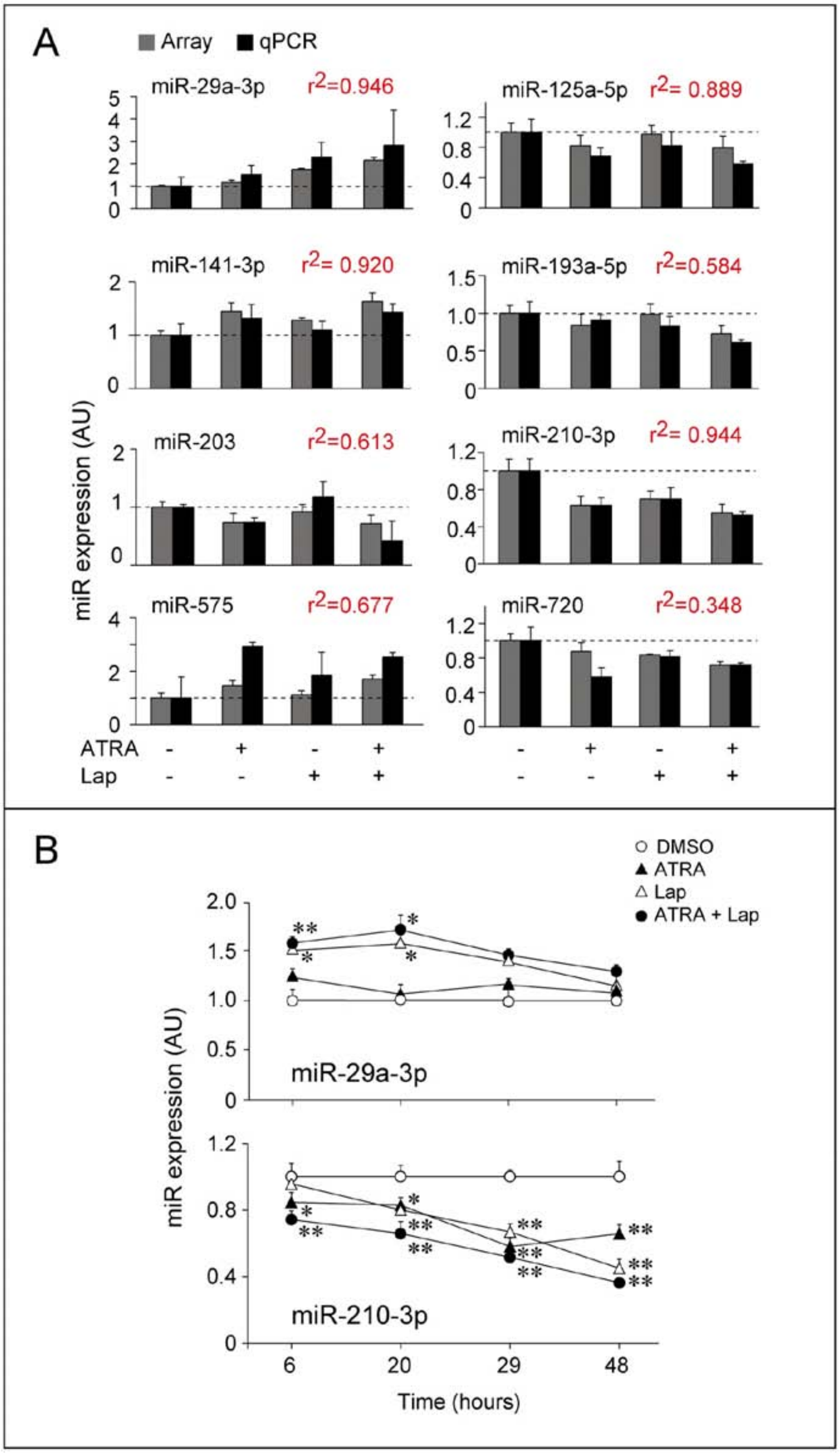

Supplementary Figure S1: Validation of miR expression by quantitative real-time PCR and time course studies on miR-29a-3p and miR-210-3p. A. The graphs illustrate the expression profiles of 8 selected miRs which were obtained with the use of miR microarrays (Array) and quantitative real-time PCR (qPCR). The numbers in red indicate the r^2 correlation values obtained after comparison of the microarray and qPCR results. Each qPCR value is the mean of 3 biological replicates ± SD. **B.** The panel shows the time-course of miR-29a-3p up-regulation and miR-210-3p down-regulation by ATRA and/or lapatinib. The results were obtained by qPCR analysis. Each result is the mean of 3 replicates ± SD. *Significantly different ($p < 0.05$, Student's *t*-test) **Significantly different ($p < 0.01$, Student's *t*-test).

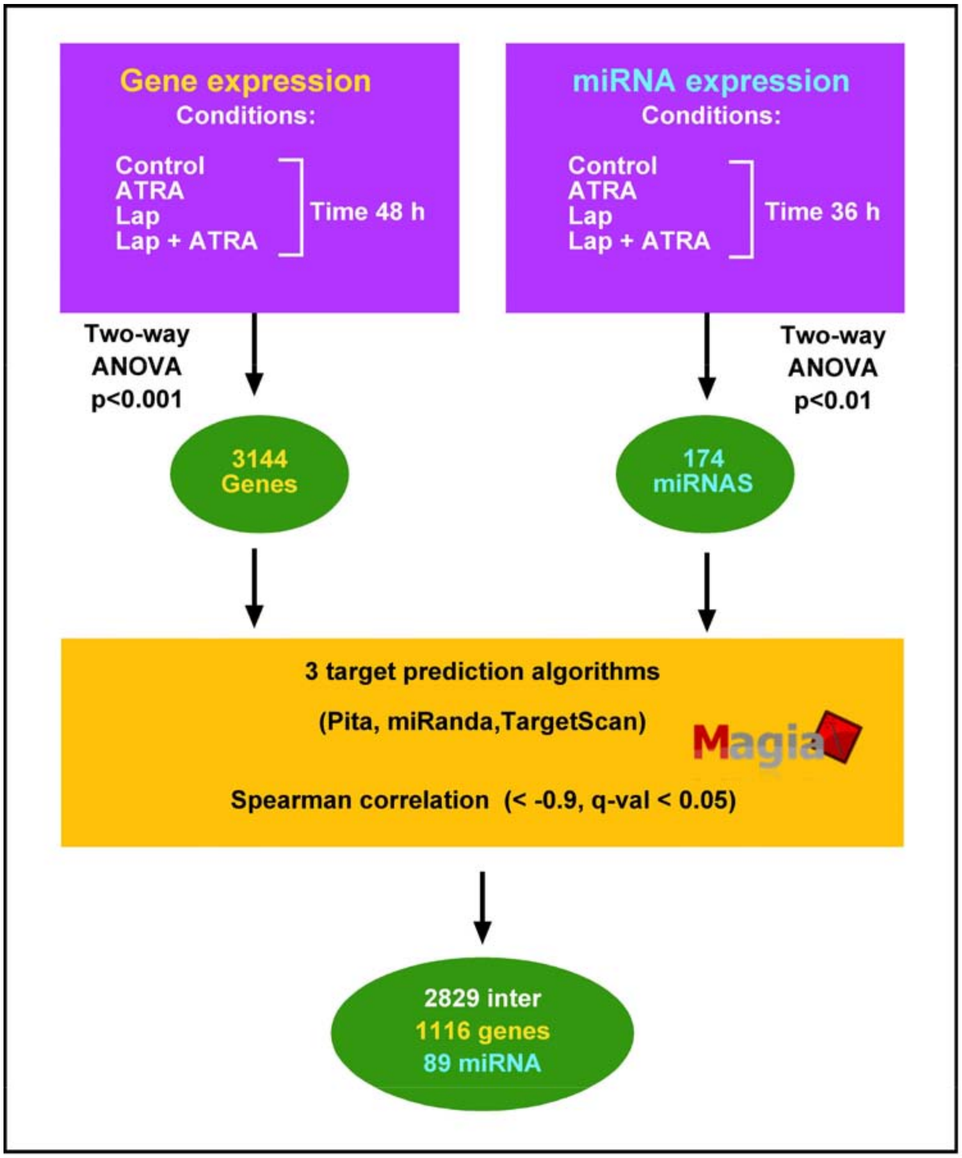

Supplementary Figure S2: Flow chart of miR and target-mRNA integrated analysis. Two active datasets from *SKBR3* cells were used for the analysis: 1) miRs selected for significant changes in expression by one of the factors in the described microarray experiments (ATRA, Lapatinib, or interaction, 36 h treatment; two-way ANOVA, $p < 0.01$); 2) mRNAs selected in the same way as in 1) from previously obtained microarray data (E-MEXP-3192; http://www.ebi.ac.uk/arrayexpress) (48 hours; $p < 0.001$). The MAGIA (MiRNA-And-Genes-Integrated-Analysis) web-tool (http://gencomp.bio.unipd.it/magia/start/) was used to predict miR/target mRNA interactions in these two datasets. The steps for the analysis were: 1) identification of putative miR/target-mRNA pairs by any one of three prediction algorithms (PITA, miRanda, TargetScan; filters set as default); 2) for each of the putative miR/target-mRNA pairs, selection of those showing a significant negative correlation between miR and mRNA-expression (Spearman correlation < – 0.9, q-value <0.05).

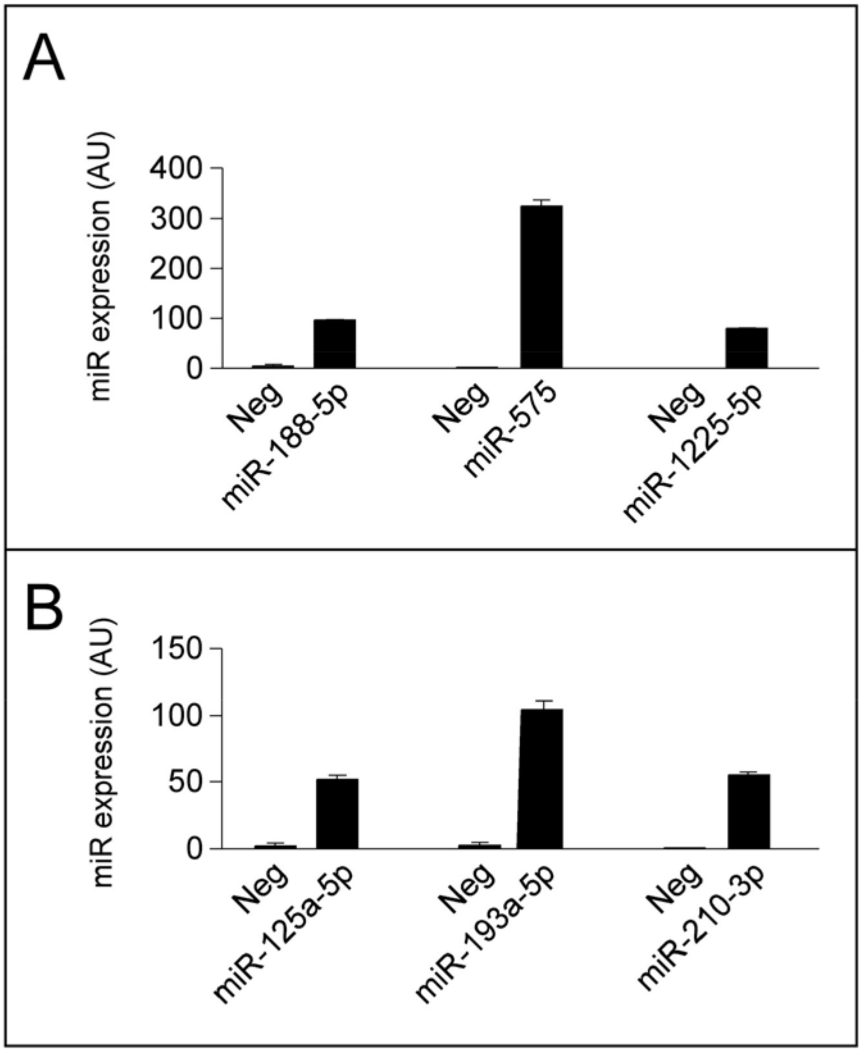

Supplementary Figure S3: Expression of *Module-1*, *Module-2* and *Module-3* miR mimics in transfected SKBR3 cells. RNA extracted from *SKBR3* cells transfected with 30 nM of the indicated miR mimics or scrambled negative control (Neg) were analyzed after 48 hours by qPCR. Specific Taqman assays were used to quantify the levels of the indicated miRs in both Neg and miR-mimic-transfected cells. Values represent the mean ± SD of 3 transfections. **A.** The panel shows the results for selected *Module-1* (miR-188-5p) and *Module-3* (miR-575 and miR-1225-5p) miRs. **B.** The panel shows the results for selected *Module-2* miRs.

Supplementary Table S1. miR profiles in SKBR3 cells following treatment with ATRA and/or lapatinib. The table lists 174 miRs whose expression is significantly altered by treatment of *SKBR3* cells with 100 nM ATRA, 100 nM Lapatinib or 100 nM of both ATRA and lapatinib for 36 hours. Columns 3–6 indicate the miR expression values measured in the 4 experimental conditions. The data are expressed in linear intensity values and they correspond to the mean of 5 biological replicates. Columns 7–10 report the *p*-values obtained after 2-way ANOVA as indicated.

Supplementary Table S2. Predicted miR/target-mRNA interactions. The table lists the miR/target-mRNA interactions predicted by the MAGIA algorithm with a Spearman correlation value < –0.9 and a *q*-value <0.05. The number in the first column indicates the module (*Module-1* to *-4*) miRs belong to. The 0 symbol is used for miRs that are not organized in any module.

Supplementary Table S3. miR and target-mRNA nodes in *Module1-4*. The table lists all the miRs and target-mRNAs belonging to the four most interconnected modules (*Module-1* to *-4*). Node = miR or target-mRNA; Degree = degree of connectivity.

Supplementary Table S4. Breast cancer cell lines characteristics. The Table lists the characteristics of the indicated cell lines. The IC_{50} and IC_{25} values (concentrations causing a 50% and 25% reduction in the number of cells) are indicated for ATRA, Lapatinib (Lap) and doxorubicin (Doxo). While the data for ATRA and Doxo were determined experimentally, the Lap data are extracted from the following article: Konecny, G.E., M.D. Pegram, N. Venkatesan, R. Finn, G. Yang, M. Rahmeh, M. Untch, D.W. Rusnak, G. Spehar, R.J. Mullin, B.R. Keith, T.M. Gilmer, M. Berger, et al. Activity of the dual kinase inhibitor lapatinib (GW572016) against HER-2-overexpressing and trastuzumab-treated breast cancer cells. Cancer Res, 2006; 66: p. 1630–9. The breast cancer cell lines were used to evaluate the effects exerted by the three compounds on the expression of miR-125a-5p, miR-193a-5p and miR-210-3p belonging to *Module-2*. The same cell lines were also used to determine the effects of the three miRs on cell growth, apoptosis and motility. TN = triple-negative; ER = Estrogen receptor.

Cell line	Phenotype	ER	HER2	ATRA IC_{50}(nM)	Lap IC_{50}(nM)	Doxo IC_{25}(nM)
MDA-MB231	Basal/TN	−	−	3, 250	18, 600	100
MDA-MB157	Basal/TN	−	−	37	6, 300	250
MDA-MB-453	*Luminal*	−	+	> 10, 000	3, 900	500
MCF-7	*Luminal*	+	−	878	7, 700	500
SKBR3	*Luminal*	−	+	72	37	100

Supplementary Table S5. Literature review for the miRs present in *Module 1–4*. The table lists the references reporting on the biological action of the miRs present in *Module-1* to *-4*. Column 3 lists the miR target-mRNAs involved.

Supplementary Table S6. Amplimers and probes. The table contains the list of the amplimers and probes used for the Taqman assays along with the list of the miR mimics used throughout the study.

miR qPCR assays	Source	Reference Code
hsa-miR-29a-3p	Exiqon	204698
hsa-miR-125a-5p	Life Technologies	002198
hsa-miR-141-3p	Exiqon	204504
hsa-miR-188-5p	Life Technologies	002320
hsa-miR-193a-5p	Life Technologies	002281
hsa-miR-203	Life Technologies	000507
hsa-miR-210-3p	Life Technologies	000512
has miR-425-3p	Exiqon	204038
hsa-miR-575	Life Technologies	001617
hsa-miR-720	Exiqon	204088
Z-30	Life Technologies	001092
**Gene expression qPCR assays**		
HIPK2	Life Technologies	Hs00179759_m1
PLCXD1	Life Technologies	Hs00895227_m1
RPLP0	Life Technologies	Hs99999902_m1
Actin	Life Technologies	Hs99999903_m1
**MiR mimics**		
hsa-miR-29a-3p	Life Technologies	PM12499
hsa-miR-125a-5p	Life Technologies	PM12561
hsa-miR-193a-5p	Life Technologies	PM11786
hsa-miR-188-5p	Life Technologies	PM12963
hsa-miR-210-3p	Life Technologies	PM10516
hsa-miR-575	Life Technologies	PM11506
hsa-miR-874-3p	Life Technologies	PM12355
hsa-miR-1225-5p	Life Technologies	PM13447
Negative Control #1	Life Technologies	AM17110
